# Extensive bilateral corneal edema 6 weeks after cataract surgery: Keratopathy due to *Asclepias physocarpa*: a case report

**DOI:** 10.1186/s12886-017-0400-z

**Published:** 2017-01-18

**Authors:** Kazuki Matsuura, Shiro Hatta, Yuki Terasaka, Yoshitsugu Inoue

**Affiliations:** 1 Nojima Hospital, 2714-1, Sesaki-machi, Kurayoshi-city, Tottori 682-0863 Japan; 2 Maejima ganka, 226, Motomachi, Tottori-city, Tottori 680-0037 Japan; 3 Tottori University, 36-1, Nishi-cho, Yonago-city, Tottori 683-504 Japan

**Keywords:** Cataract surgery, Endophthalmitis, Asclepias physocarpa, Case report

## Abstract

**Background:**

Surgeons may be unaware of the ability of plant toxins to cause corneal damage. Therefore, corneal damage following intraocular surgery due to plant toxins may be misdiagnosed as postoperative infection.

**Case presentation:**

A 74-year-old man presented with hyperemia and reduced visual acuity in both eyes 6 weeks after uneventful cataract surgery. We observed extensive hyperemia and corneal stromal edema with Descemet’s folds in both eyes.

After obtaining a detailed patient history, we diagnosed plant toxin-induced corneal edema due to *Asclepias physocarpa*, which can induce corneal edema by inhibiting the Na^+^/K^+^ ATPase activity of the corneal endothelium.

Antimicrobial and steroid eye drops and an oral steroid were prescribed accordingly. Symptons began to improve on day 3 and had almost completely resolved by day 6. At 1 month, the patient had fully recovered without any sequelae.

**Conclusion:**

The correct diagnosis was possible in the present case as symptoms were bilateral and the patient was able to report his potential exposure to plant toxins. However, if the symptoms had been unilateral and the patient had been unaware of these toxins, he may have undergone unnecessary surgical interventions to treat non-existent postoperative endophthalmitis.

## Background

Bacterial endophthalmitis is a clinically significant condition that should be considered in patients complaining of vision loss accompanied by acute extensive inflammation in the anterior segment of the eye within a few weeks of cataract surgery. Frequent examinations are generally required in addition to antibiotic treatment, with emergency surgery often required. We experienced case of corneal edema accompanied by extensive inflammation occurring 6 weeks after uneventful bilateral cataract surgery. In the present case, we successfully diagnosed plant toxin-induced corneal edema [1–3] by obtaining a detailed patient history and thereby avoiding unnecessary surgical interventions.

## Case presentation

**Fig. 1:**
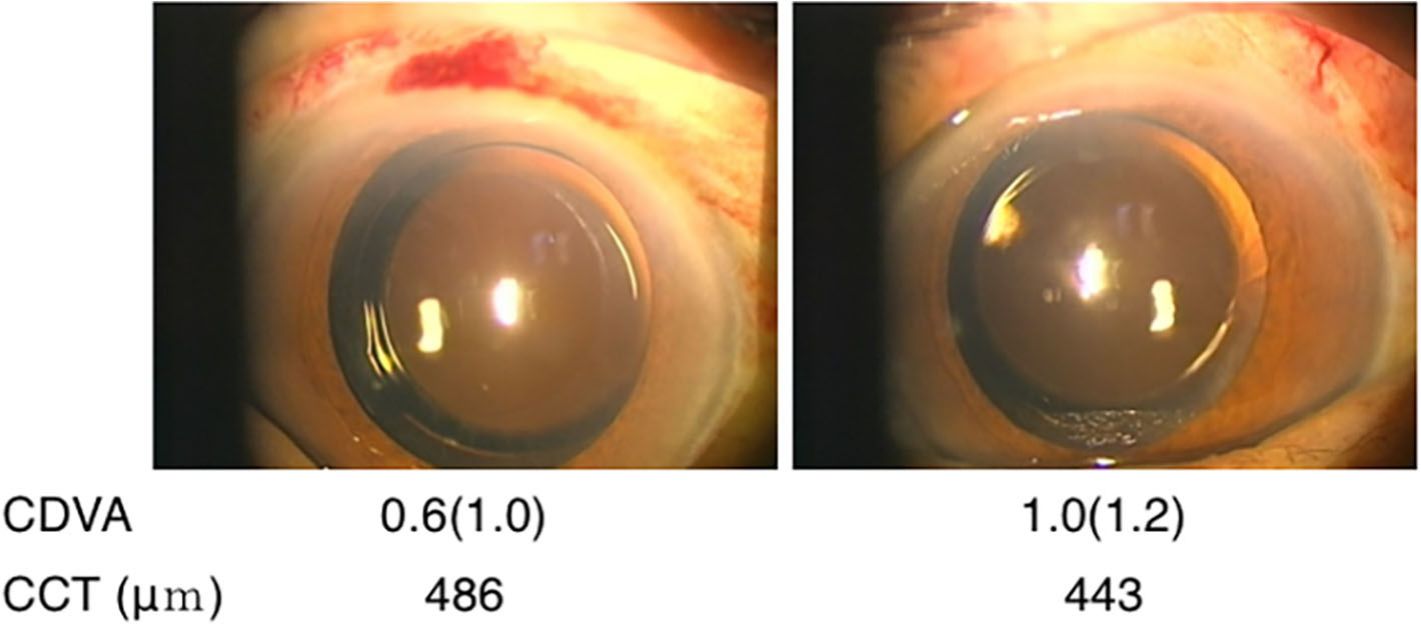
Uneventful cataract surgeries were conducted in both eyes. CCT: central corneal thickness, CDVA: corrected distant visual acuity

**Fig. 2:**
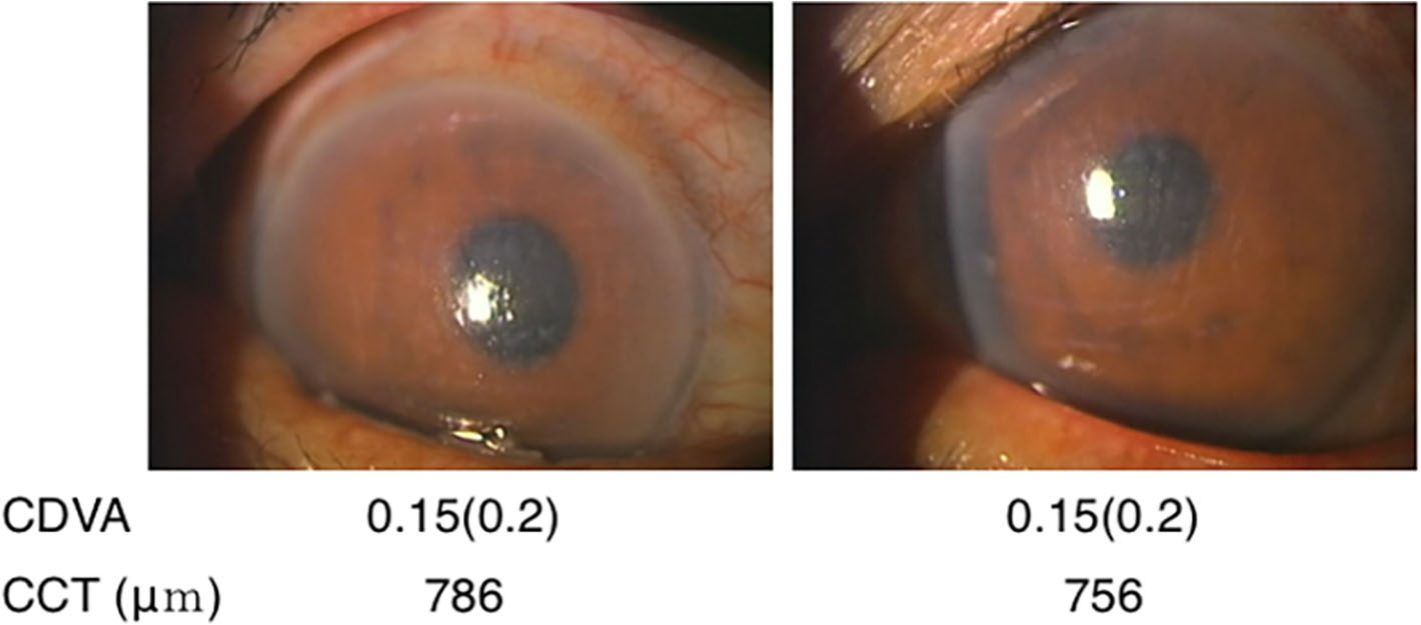
Conjunctival injection and corneal edema with Descemet’s folds (day 1)

A 74-year-old man with no history of uveitis, keratitis, or allergic eye disease underwent uneventful bilateral cataract surgery with one day interval in August 2015. His best corrected distant visual acuity (CDVA) following surgery was 1.0 and 1.2 in the right and left eyes, respectively (Fig. 1). Intraocular pressures and the number of corneal endothelial cells (2301/mm^2^ in the right eye and 2171/mm^2^ in the left) were within the normal range. The patient reported hyperemia and reduced visual acuity in both eyes one evening at 6 weeks postoperatively and visited Maejima eye clinic the following day. On examination, extensive hyperemia and corneal stromal edema with Descemet’s folds were observed in both eyes. His CDVA was 0.2 in both eyes, and central corneal thicknesses were 786 μm and 756 μm in the right and left eyes, respectively (Fig. 2). Intraocular pressure measurements and detailed observations of the anterior chamber were not possible due to the presence of corneal edema. We first suspected bacterial endophthalmitis; however, we considered this an atypical case due to the simultaneous development of symptoms in both eyes 6 weeks postoperatively. No apparent vitreous opacity, retinal hemorrhage, or vasculitis was evident. After obtaining a detailed patient history, it was apparent the patient had performed gardening work during the day of onset. The patient had been engaged in the cultivation and sale of ornamental plants and had heard of eye toxicity on exposure to certain plants. We requested the patient list the plants he had handled and conducted a literature search. His clinical symptoms were consistent with those reported for corneal damage due to *Asclepias physocarpa* ^1^. We therefore diagnosed plant toxin-induced corneal edema accordingly. His eyes were initially thoroughly rinsed with normal saline. Topical levofloxacin 1.5% (6 times/day) and bethamethasone 0.1% (6 times/day) were administered, and he was re-evaluated daily on an outpatient basis. Small doses (10 mg) of oral steroids were added as the presence of retinal and vitreal inflammation could not be completely excluded.

**Fig. 3:**
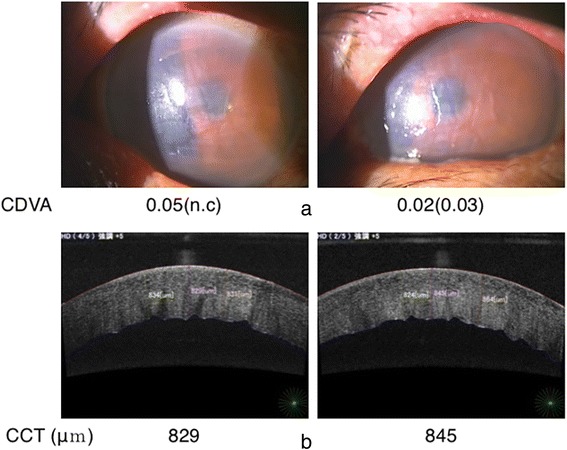
**a**. Conjunctival injection and severe corneal edema with Descemet’s folds (day 2). **b**. Anterior segment optical coherence tomography (SS-1000, TOMEY Inc, Aichi, Japan). Central corneal thickness measurements were 829 μm and 845 μm for the right and left eyes, respectively (day 2). CCT: central corneal thickness, CDVA: corrected distant visual acuity n. c: non corrigunt

The next day, conjunctival injection and corneal edema had increased, and his CDVA had decreased to 0.05 and 0.03 in the right and left eyes, respectively (Fig. 3). Corneal edema began to improve on day 3 and had almost completely resolved by day 6. The oral steroid was ceased immediately after recovery of the visibility (day5).

**Fig. 4:**
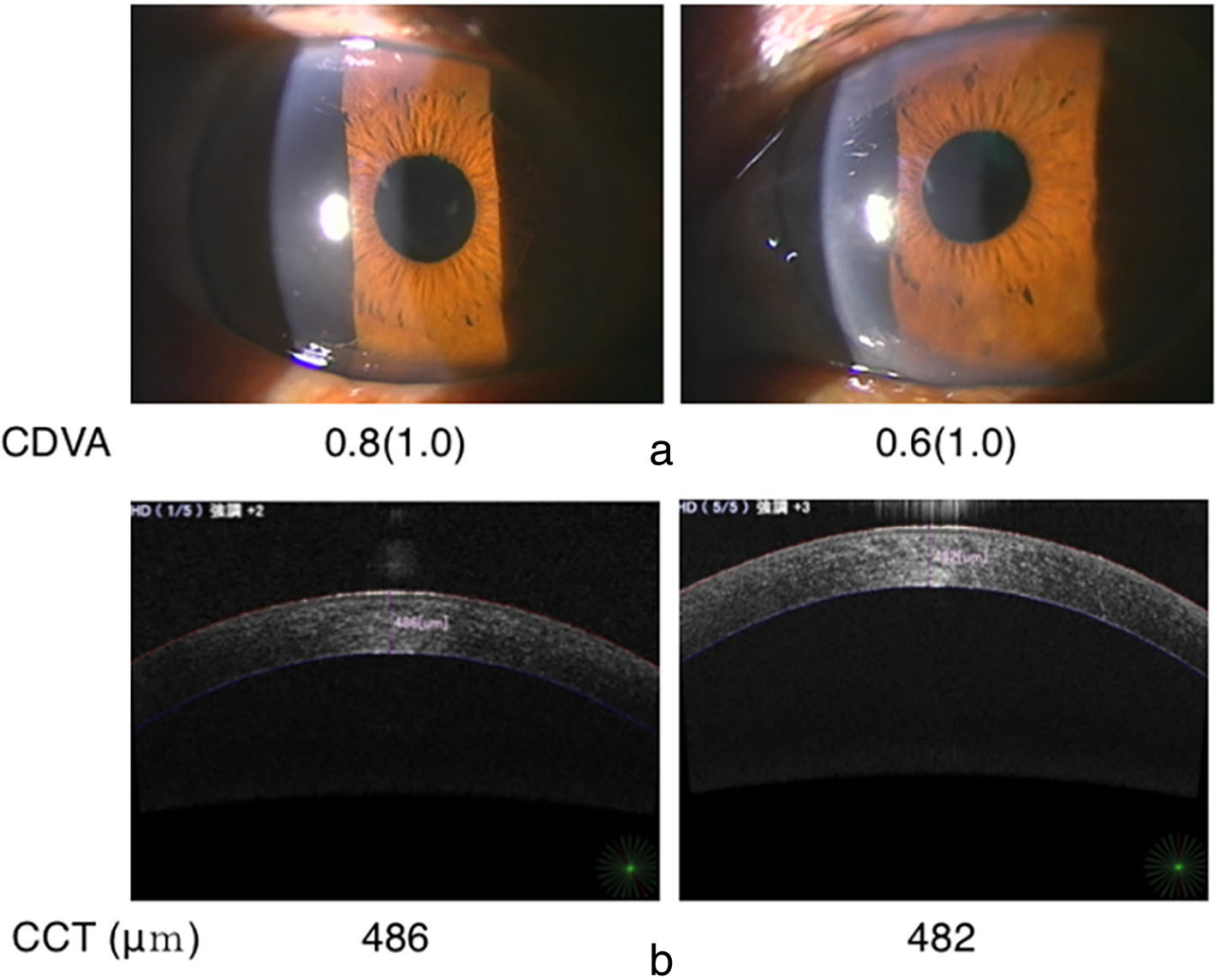
**a**. Almost complete resolution of conjunctival injection and corneal edema (day 8). **b**. Anterior segment optical coherence tomography. Central corneal thickness measurements were normalized at 486 μm and 482 μm for the right and left eyes, respectively (day 8). CCT: central corneal thickness, CDVA: corrected distant visual acuity

On day 8, his CDVA had recovered to 1.0 in both eyes (Fig. 4). No clinical signs were evident at 1 month. The intraocular pressure and number of corneal endothelial cells (2171/mm^2^ in the right eye and 2247/mm^2^ in the left) were within the normal range.

## Discussion

**Fig. 5:**
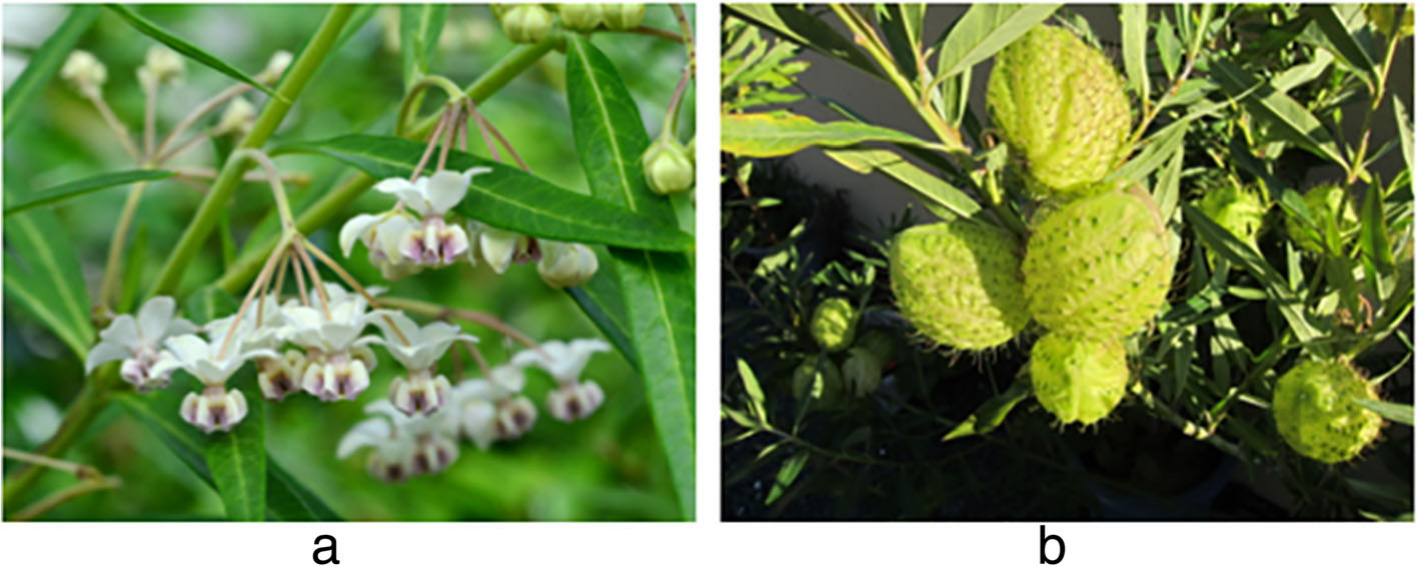
**a**. Flower and leaves of *Asclepias physocarpa.* **b**. Follicle of *A. physocarpa*

*A. physocarpa*, commonly known as tropic and subtropic milkweed, is a plant species native to tropical America and belongs to the genus *Asclepias* of the family *Apocynaceae* (Fig. 5). The plants of the *Asclepias* genus are widely distributed as horticultural or ornamental plants in Japan and other countries, and latex from their stems has been shown to contain toxic components termed cardenolides. To our knowledge, only a few cases of cardiolides toxicity have been previously reported [1–3], and general ophthalmologists are unaware of these plants. In corneal endothelial cells, the Na^+^/K^+^ ATPase actively transports Na^+^ from inside of the cells into the anterior chamber to create an osmotic pressure gradient; thereby resulting in the active transport of water from inside the corneal stroma to the anterior chamber. The corneal stromal edema obsereved in the present case was attributable to corneal endothelial dysfunction caused by suppression of the Na^+^ active transport by cardenolides in the stem latex, thereby inhibiting the Na^+^/K^+^ ATPase [4].

Chakraborty et al. reported a case of *A. curassavica* exposure in which the patient rapidly attained remission with the use of artificial tear eye drops only [2]. Amiran et al. reported a case of *A. fruticosa* exposure in which the clinical signs showed marked rapid improvement after the use of 0.1% topical dexamethasone [3]. Pina et al. reported a case of *A. physocarpa* exposure in which almost complete resolution was obtained using topical dexamethasone, ofloxacin, and artificial tears. Pina et al. suggested the possibility of abnormal endothelial morphology as a sequelae at 6 months follow-up, although the cell count was within the normal range (2119 cells/mm^2^). The use of topical steroids may increase the activity of the Na^+^/K^+^ pump in corneal endothelial cells [5] and thus have utility in the treatment of plant-induced corneal edema. Therefore, we modelled our treatment on the approach reported by Pina et al. [1], with the patient almost completely recovering within 6 days. Our patient fully recovered without any sequelae including any damage to corneal endothelial cells.

Few plants such as those in the genus *Asclepias* have been reported to cause corneal damage. In a report of 7 cases of eye damage caused by *Euphorbia* plants, aggressive antibacterial and anti-inflammatory treatments were considered necessary as the patients presented with defects in the corneal epithelium and stromal edema, iritis, and other symptoms; with one case ultimately leading to blindness [6].

In a report on 29 cases of eye damage due to *Calotropis procera*, the patients recovered within 3–14 days with maintenance of corneal transparency. Although visual acuity also recovered satisfactorily in the majority cases, endothelial cell counts were decreased in 74% of these cases. Epithelial defects, iritis, and increased ocular pressure were observed in 10, 31, and 24% of cases, respectively [7].

In the present case, the patient did not remember any direct entry of latex into his eyes. As in the present case, corneal damage may be caused by contact between the eyes and hands that have been contaminated with latex, even without direct entry of latex droplets into the eyes. Therefore, physicians should proactively determine the involvement of plant toxins and conduct a detailed patient interview in such cases.

## Conclusion

Although plants of the genus *Asclepias* are widely distributed, the number of the reports of ophthalmic disease due to plant toxins is surprisingly low. This may be attributable to the unfamiliarity of ophthalmologists to plant-based toxicity. In the present case, the correct diagnosis was possible as symptoms were bilateral and the patient could report his exposure to plant toxins. However, if the symptoms had been unilateral or developed more immediately postoperatively, or if the patient had been unaware of plant toxins, he may have undergone unnecessary surgical interventions. Although plant toxin-induced corneal damage is rarely encountered, all surgeons should be aware of this significant clinical condition.

## Abbreviation

CDVA: Corrected distant visual acuity
